# Cognitive Impairment Is Independently Associated with Non-Adherence to Antithrombotic Therapy in Older Patients with Atrial Fibrillation

**DOI:** 10.3390/ijerph16152698

**Published:** 2019-07-29

**Authors:** Hyun-Joo Seong, Kyounghoon Lee, Bo-Hwan Kim, Youn-Jung Son

**Affiliations:** 1 Red Cross College of Nursing, Chung-Ang University, Seoul 06974, Korea; 2 College of Medicine, Division of Cardiology, Gachon University, Incheon 21565, Korea; 3 College of Nursing, Gachon University, Incheon 21936, Korea

**Keywords:** atrial fibrillation, aged, medication adherence, cognitive dysfunction, health literacy

## Abstract

Atrial Fibrillation (AF) patients could reduce their risk of stroke by using oral antithrombotic therapy. However, many older people with AF experience cognitive impairment and have limited health literacy, which can lead to non-adherence to antithrombotic treatment. This study aimed to investigate the influence of cognitive impairment and health literacy on non-adherence to antithrombotic therapy. The study performed a secondary analysis of baseline data from a cross-sectional survey of AF patients’ self-care behaviors at a tertiary university hospital in 2018. Data were collected from a total of 277 AF patients aged 65 years and older, through self-reported questionnaires administered by face-to-face interviews. Approximately 50.2% of patients were non-adherent to antithrombotic therapy. Multiple logistic regression analysis revealed that cognitive impairment independently increased the risk of non-adherence to antithrombotic therapy (odds ratio = 2.628, 95% confidence interval = 1.424–4.848) after adjustment for confounding factors. However, health literacy was not associated with non-adherence to antithrombotic therapy. Cognitive impairment is a significant risk factor for poor adherence to antithrombotic therapy. Thus, health professionals should periodically assess both cognitive function after AF diagnosis and adherence to medication in older patients. Further studies are needed to identify the factors that affect cognitive decline and non-adherence among AF patients.

## 1. Introduction

Atrial fibrillation (AF) is the most common type of arrhythmia and prevalent among older people [1,2]. About 70% of patients with AF are between the ages of 65 and 85 years [3,4]. Although AF itself is not usually life-threatening, mortality is higher and disability increases when stroke occurs in association with AF [5,6]. A number of studies have shown that older adults with AF have a four- to five-fold higher risk of stroke [6,7,8]. Therefore, stroke prevention in older patients with AF is an immense public health concern.

In particular, for the majority of elderly patients, long-term use of antithrombotic medications is required to prevent the risk of stroke, which can be classified into antiplatelet agents and anticoagulants [3]. Antiplatelet agents, such as aspirin, inhibit platelet aggregation and anticoagulants, such as warfarin or non-vitamin K antagonist oral anticoagulants (NOACs), prevent thrombus by interfering with hemostasis [9]. In particular, the NOACs have several benefits over the warfarin. They do not require routine blood testing for international normalized ratio (INR) monitoring because they are administered at a fixed daily dose [9,10]. But adverse events, especially the risk of bleeding, remain an issue. Even though NOAC’s less burdensome treatment can lead to better adherence, older patients are less likely to adhere to antithrombotic therapy, due to risk of bleeding [10,11]. According to prior studies, approximately one fourth of AF patients stopped taking anticoagulants after the first year [7,12]. Non-adherence to antithrombotic therapy can lead to frequent hospital admissions and increased mortality in the AF population [13]. Particularly, older adults with chronic diseases are less likely to adhere to medication compared to younger people [14,15].

Cognitive impairment or decline is a common condition in AF, which influences a patient’s ability for self-care and performing simple tasks of daily living [16,17]. Numerous studies have shown a link between AF and cognitive impairment, likely due to thromboembolism or chronic cerebral hypo-perfusion [1,4]. It is estimated that patients with AF have around two to three times higher risk of cognitive impairment and dementia, compared to those who have a normal sinus rhythm [1,7,18]. Medication adherence involves several cognitive functions, including accessing and scheduling medications, and understanding, remembering, and following instructions, all of which may be affected by cognitive impairment [19]. Several studies suggest that AF patients with cognitive impairment have poorer anticoagulation adherence, compared to those without cognitive impairment [8,14,17]. However, there is a lack of data regarding these issues, especially in Korea.

Health literacy is the ability to access, read, understand, and use basic health information and services needed to make informed decisions [20,21]. Inadequate health literacy has been related to lower medication adherence, as well as a poorer disease status and increased health costs [21,22]. Previous studies have demonstrated that individuals receiving oral anticoagulation have significantly less knowledge about the purpose of anticoagulation, which results in non-adherence to antithrombotic therapy [22,23]. Nevertheless, evidence regarding the association between health literacy and antithrombotic therapy for stroke prevention in AF remains limited. 

Therefore, we aimed to investigate associations between cognitive impairment, health literacy, and non-adherence to antithrombotic therapy, in AF patients 65 years and older receiving medical service in outpatient clinics. 

## 2. Methods

### 2.1. Study Design and Participants

This was a cross-sectional study using secondary data analysis. The primary data exploring AF patients’ self-care behaviors [24] were collected from February to August in 2018, using a structured questionnaire to collect data, in a tertiary general hospital in Korea.

The inclusion criteria were: (1) Adults aged 65 years or over, (2) outpatients who had been undergoing antithrombotic therapy for at least 1 year since physician-confirmed diagnosis of AF, and (3) individuals who were fully capable of communication. Exclusion criteria were as follows: (1) Patients with transient AF, (2) those who were taking antithrombotic medications for purposes other than AF, (3) patients who had severe comorbidities, such as advanced cancer and chronic obstructive pulmonary disease, and (4) patients with a past history of stroke and cognitive problems, such as mild cognitive impairment or dementia before AF diagnosis.

Sample size was computed using G*Power 3.1.9.2 software (Universität Düsseldorf, Düsseldorf, Germany). For a moderate-effect size of 1.5 (odds ratio), significance level of 0.05, power of 0.90, and a two-tailed test for multiple logistic regression, the required minimum sample size was 275. Therefore, our study population of 277 participants satisfied the minimum sample requirement for these analyses.

### 2.2. Instruments

#### 2.2.1. Socio-Demographic and Disease-Related Characteristics of Patients

In accordance with prior studies [2,5,25], socio-demographic characteristics, including age, gender, education level, employment status, and marital status, were considered. In addition, disease-related characteristics, including time since diagnosis with AF, type of AF, CHA_2_DS_2_-VASc scores, HAS-BLED scores, comorbidity, and type of antithrombotic agents, were assessed. 

CHA_2_DS_2_-VASc scores assess the risk of and are an important tool for the prevention of stroke, which is a major goal of antithrombotic therapy [26]. CHA_2_DS_2_-VASc scores are the sum of scores for congestive heart failure (1 point); hypertension (1 point); age (≥75 years: 2 points, 65–74 years: 1 point); diabetes mellitus (1 point); stroke, transient ischemic attack, and thromboembolism (2 points); sex (female: 1 point); and vascular disease (1 point), totaling a maximum score of 9. Scores are interpreted as low risk (0 points), intermediate risk (1 point), or high risk (≥2 points) of stroke. A higher CHA_2_DS_2_-VASc score indicates a higher risk for stroke [27].

HAS-BLED scores assess the risk of hemorrhage, and consider hypertension (1 point), kidney or liver dysfunction (1 point each), stroke (1 point), bleeding history or tendency (1 point), unstable INR (1 point), old age (>65 years: 1 point), and administration of medications that increase bleeding tendency or alcohol consumption (1 point each) as bleeding risk factors [26]. The maximum score is 9, and HAS-BLED scores are interpreted as low risk (0 point), intermediate risk (1–2 points), or high risk (≥3 points) of bleeding. A higher score indicates a greater possibility of hemorrhage [27].

#### 2.2.2. Cognitive Function

Cognitive functions were measured using the K-MMSE, the Korean version of the MMSE developed by Folstein et al. [28], translated and validated by Kang et al. [29] with the original items retained. The K-MMSE is widely used, as various cognitive functions can be quickly and conveniently measured within 5–15 minutes [29]. The K-MMSE consists of 30 items in seven categories: Time orientation (5 items), spatial orientation (5 items), working memory (3 items), concentration/attention (5 items), memory recall (3 items), language (8 items), and visuospatial abilities (1 items). The scores range from 0 to 30, with a lower score indicating lower cognitive function. The cutoff score was set to 24, based on previous studies [19,30] where a score of less than 24 indicated impaired cognitive function.

#### 2.2.3. Health Literacy

Health literacy was measured using the Three Brief Health Literacy Screeners originally developed by Chew et al. [3]. This tool consists of three items, each rated on a 5-point scale (0–4). With item one scored in reverse, the total score ranges from 0 to 12. A higher total score indicates a greater ability to understand and utilize health information [31]. According to a previous study [32], a score below 6 indicates inadequate health literacy, a score of 7–10 indicates marginal health literacy, and a score of 11–12 indicates adequate health literacy. The reliability of the tool in the present study was high, with Cronbach’s α = 0.830.

#### 2.2.4. Adherence to Antithrombotic Therapy

Adherence to antithrombotic therapy was measured with a single item developed by Wu et al. [33]. A single-item question showed similar results to that of counting the number of drugs actually taken [34], and was reported to significantly predict patient mortality and readmission rates (IRR = 0.84, *p* < 0.001). A six-point Likert scale was used for the question “In the past week, have you forgotten to take your antithrombotic medication for various reasons?” (1 = “Always”, 2 = “Almost always”, 3 = “Frequently”, 4 = “Occasionally”, 5 = “Sometimes”, 6 = “Never”). Answers from 1 to 5 indicate non-adherence, and answer 6 indicates adherence [33].

### 2.3. Ethical Considerations and Data Collection

This present study was reviewed and approved by the Institutional Review Board (approval number 1041078-201712-HRZZ-234-01). Participants were informed that there would be no penalty for refusing or withdrawing from participation at any point during the study, and that their anonymity would be maintained. Informed written consent was obtained from all patients. Face-to-face interviews using a pretested, structured questionnaire were carried out by two trained research assistants. 

### 2.4. Data Analysis

Data were analyzed using SPSS/WIN 23.0 software (IBM Corp., Armonk, NY, USA). Data are presented as mean ± standard deviation (SD) for normally distributed continuous variables, and frequencies and percentages for categorical variables. Differences between groups were analyzed using the Pearson’s chi-squared test. To determine factors independently associated with non-adherence to antithrombotic therapy in older adults with AF, multiple logistic regression analysis was performed with variables that were significant in univariate analysis. A two-sided *p* value of <0.05 was used as the cutoff for statistical significance.

## 3. Results

### 3.1. Socio-Demographic and Disease Characteristics of Patients with AF

As shown in Table 1, mean age of the 277 study participants was 74.2 ± 7.2 years. The majority of participants were aged 70–79 years (45.5%, *n* = 126). Men accounted for 59.2% of participants (*n* = 164). About 34.3% (*n* = 95) had elementary school education or below. 

With regard to disease-related characteristics, the mean time since diagnosis with AF was 7.9 ± 6.9 years. Approximately, 61.4% (*n* = 170) had paroxysmal AF and 35% (*n* = 97) had persistent AF. The mean risk of stroke (CHA_2_DS_2_-VASC scores) and hemorrhage (HAS-BLED scores) were 3.8 ± 1.6, and 3.2 ± 0.9, respectively. About 44.0% (*n* = 122) of the participants were taking aspirin, 35.7% (*n* = 99) were taking warfarin, and 28.5% (*n* = 69) were taking NOACs (Table 1). 

### 3.2. Level of Cognitive Function, Health Literacy, and Adherence to Antithrombotic Therapy

The mean cognitive function score was 25.8 ± 3.6 out of 30. Based on a cutoff score of 24, 28.9% (*n* = 80) had cognitive impairment (<24). The mean health literacy score was 7.9 ± 3.5 out of 12. Most participants had marginal (45.5%, *n* = 126) or inadequate health literacy (28.1%, *n* = 78). The rate of non-adherence to antithrombotic therapy was around 50.2% (*n* = 139) (Table 2).

### 3.3. Differences in Adherence to Antithrombotic Therapy according to General Characteristics, Cognitive Function, and Health Literacy

Adherence to antithrombotic therapy significantly differed according to age (*p* = 0.001), education level (*p* = 0.001), job (*p* = 0.017), spouse (*p* = 0.001), CHA_2_DS_2_-VASc score (*p* = 0.012), cognitive function (*p* < 0.001), and health literacy (*p* < 0.001). However, there were no significant differences in adherence to antithrombotic therapy according to gender, year after diagnosis of AF, type of AF, risk of hemorrhage, comorbidities, and type of antithrombotic agents (Table 3). 

### 3.4. Factors Influencing Non-Adherence to Antithrombotic Therapy in Older Patients with AF

A shown in Table 4, logistic regression analysis indicated that non-adherence to antithrombotic therapy is associated with cognitive impairment alone (odds ratio = 2.628, 95% confidence interval = 1.424–4.848), after adjustment for factors including age, education level, job, spouse, and CHA_2_DS_2_-VASc score. However, health literacy was not associated with non-adherence to antithrombotic therapy.

## 4. Discussion

Older adults with AF are undoubtedly at higher risk of heart failure, myocardial infarction, stroke, dementia, and thromboembolism with fatal complications [1,6]. Thus, antithrombotic therapy, which effectively lowers these risks, is important for maintaining optimal health and quality of life in AF patients [9]. 

In our study, about 50.2% of participants showed non-adherence to antithrombotic therapy. This is similar to previous studies reporting that more than 50% of patients with chronic disease show non-adherence to medication [10,32]. Considering the prevalence of poor adherence, health professionals should be aware of whether patients actually take their medication or not, when evaluating the impact of medication on health outcomes. However, antithrombotic therapy non-adherence rates, as reported by Jankowska-Polańska et al. [35] and Obamiro et al. [9], were 65.7% and 45.1%, respectively. This contrast appears to be due to differences in instrumentation, and cut-off scores in non-adherence assessment tools. Most importantly, the reason for patients’ suboptimal adherence to medication should be identified. Furthermore, NOACs do not require recurrent monitoring of the anticoagulant effect with dose adjustment [10]. Thus, NOACs may result in increased patient adherence compared with warfarin. However, due to their short half-life, the efficacy of NOACs may disappear quickly if those are missed [9,11]. Consequently, medication adherence is also important for NOACs as well as warfarin. Our study showed that there was no significance between type of antithrombotic medication and adherence to medication. To gain a better understanding of medication adherence rates of NOACs versus warfarin, longitudinal studies with a larger sample are needed. 

First and foremost, our main result demonstrated that cognitive impairment is a significant risk factor for non-adherence to antithrombotic therapy in older adults with AF, after adjustment for confounding factors. This finding is similar to the results of previous studies, showing that cognitive function is associated with medication adherence among AF patients [7,35,36]. Jankowska-Polańska et al. [35] found that 48.1% of the non-adherence group had cognitive impairment, and that the non-adherence group had a lower cognitive function score than the adherence group. Some studies using INR to measure adherence also found that patients with cognitive impairment maintain an inadequate INR range [37]. This finding is related to the hypothesis that mechanisms linking AF to cognitive decline include altered hemodynamics, resulting in cerebral hypoperfusion [16,19]. Cognitive dysfunction due to AF in older adults impairs planning and organizing abilities, and medication-taking behavior [9]. Thus, cognitive functioning tests should be considered for AF patients who are beginning antithrombotic therapy for the first time in a healthcare setting [11]. Particularly, elderly AF patients’ cognitive functions should be examined prior to providing medication education and care. Furthermore, our findings support the feasibility of long-term interventions that improve medication adherence, based on patients’ cognitive function, to prevent cognitive decline in older adults with AF. 

In contrast, the results of the present study are not consistent with those of the prospective study by Horstmman et al. [5], showing that cognitive impairment is not a risk factor associated with non-adherence to oral anticoagulation in stroke with AF. In addition, a recent study in a general adult population similarly found no association between cognitive impairment and adherence to cardiovascular medication, after adjustment for a range of potential confounders [19]. This discrepancy between our results and previous results might be explained by the participants’ ages, different measures and definitions of cognitive impairment, and medication adherence. In terms of participant age, the trend may be related to a general decrease in cognitive function scores with increasing age. Based on the cut-off score of 24, our mean cognitive function score of 25.81 was lower than those found in previous studies by Bellomo et al. [4] and Serpytis et al. [38], using the same tool (26.9 and 27.5, respectively). However, this score was higher than that reported by Mizrahi et al. [39] (MMSE score of 20.6). Accordingly, a standardized and objective assessment is needed to clarify the association between cognitive impairment and medication non-adherence in AF. 

With regard to health literacy, our regression analysis showed that health literacy did not affect non-adherence to antithrombotic therapy. Similarly, Roll et al. [22] reported that health literacy did not influence adherence to antithrombotic therapy in AF patients. In our study, 28.1% of the participants had inadequate health literacy, similar to the rate (20.4%) found in the study by Reading et al. [23], using the same tool and study population. Although the prevalence rate of inadequate health literacy in our study was not relatively low compared to previous studies in chronic disease [14,32], the rate of inadequate health literacy was higher in the non-adherent group compared to those in the adherent group in our univariate analysis. This implies that there is possibly a significant association between health literacy and antithrombotic medication adherence in older adults with AF. Thus, patient-centered education programs should be designed to enhance health literacy in older adults with AF. 

In addition, our univariate analysis showed that non-adherence to anti-thrombotic therapy might be related to older age, low education level, no job, no spouse, and higher stroke risk (CHA_2_DS_2_-VASc score). Older age and education level can affect patients’ ability to comprehend medication-related information [21,22]. In accordance with our study, Ferguson et al. [25] found that people without a spouse showed higher non-adherence rates. Non-adherence may be higher among older adults who live alone because there is no one to remind them to take their medications, having to rely on themselves [15]. Having and maintaining a job may require the individual to be in good health. In contrast, health risks, such as AF with antithrombotic therapy, may lead to job loss or lack of performance, and poor self-care with medication non-adherence [12,40]. In agreement with our study, Deshpande et al. [41] found that stroke risk in AF patients was influenced by medication non-adherence. Accordingly, more comprehensive research is needed to describe factors influencing antithrombotic therapy non-adherence, considering the importance of overall geriatric assessment. 

Older AF patients more often had persistent or permanent AF compared with younger patients [4]. Progression of AF from paroxysmal to persistent is faster in older patients [1]. Older AF patients also had a higher prevalence of comorbidities, including hypertension, coronary artery disease, heart failure, and other chronic diseases, which lead to polymedication [35]. These conditions may be associated with the increased risk of cognitive impairment. However, the potential relationships between AF, comorbidities, and cognitive impairment are not fully illuminated. Therefore, future studies should address causal relationships between these factors for identifying possible risk factors on medication non-adherence among elderly patients with AF. This cross-sectional observational study demonstrated the impact of cognitive impairment on non-adherence to antithrombotic therapy. Interestingly, a recent meta-analysis has reported the protective effects of NOACs on cognitive functions [42]. Therefore, long-term observational studies are necessary for obtaining a bidirectional relationship between cognitive impairment and antithrombotic non-adherence in AF patients.

Our study has some limitations. First, this study was a cross-sectional survey that used a convenience sample of participants at a single hospital. Second, self-reported assessment tools for cognitive assessment, health literacy, and medication adherence were adopted. For this reason, over-reporting or under-reporting could be influenced by social desirability. Thus, in addition to subjective assessment tools, objective assessment tools should also be considered.

## 5. Conclusions

Our study highlights that cognitive impairment is independently associated with non-adherence to antithrombotic therapy in older people with AF. Therefore, health professionals should be aware that periodical assessment of cognitive function before starting antithrombotic therapy is important and relevant to patient illness trajectories. Furthermore, if a patients shows cognitive decline or impairment, health professionals have to encourage patients and caregivers to realize the importance of antithrombotic medication adherence, and consider proper strategies to optimize the cognitive function of elderly AF patients. 

A simplified and validated screening tool for medication adherence, as well as electronic monitoring devices providing objective measurements, such as pill counts, are required for older patients with comorbidities. Longitudinal studies are needed to investigate the causal effect of cognitive impairment on non-adherence to antithrombotic agents.

## Figures and Tables

**Table 1:** Socio-demographic and disease characteristics of patients with atrial fibrillation (*n* = 277).

Characteristics	Categories	*n* (%)	Mean ± SD
Age (years)	65–70	89 (32.1)	74.2 ± 7.2
	70–79	126 (45.5)	
	≥80	62 (22.4)	
Gender	Men	164 (59.2)	
	Women	113 (40.8)	
Education	Below elementary school	95 (34.3)	
	Middle school	69 (24.9)	
	High school	77 (27.8)	
	College	36 (13.0)	
Job	Yes	58 (20.9)	
Spouse	Yes	190 (68.6)	
Years after diagnosis of AF	1–2	40 (14.4)	7.9 ± 6.9
	3–4	42 (15.2)	
	≥5	195 (70.4)	
Type of AF	Paroxysmal	170 (61.4)	
	Persistent and permanent	107 (39.6)	
CHA_2_DS_2_-VASc score	1	21 (7.6)	3.8 ± 1.6
	≥2	256 (92.4)	
HAS-BLED score	1–2	53 (19.1)	3.2 ± 0.9
	≥3	224 (80.9)	
Hypertension	Yes	221 (79.8)	
Diabetes	Yes	79 (28.5)	
Coronary artery disease	Yes	90 (32.5)	
Heart failure	Yes	110 (39.7)	
Stroke	Yes	57 (20.6)	
Aspirin	Yes	122 (44.0)	
Warfarin	Yes	99 (35.7)	
NOACs	Yes	69 (28.5)	

SD, standard deviation; AF, atrial fibrillation; NOACs, non-vitamin K antagonist oral anticoagulants.

**Table 2:** Level of cognitive function, health literacy, and adherence to antithrombotic therapy *(n* = 277).

Variables (Cut-Off)	*n* (%)	Min–Max	Mean ± SD
Cognitive function		14–30	25.8 ± 3.6
Impaired (<24)	197 (71.1)		
Normal (≥24)	80 (28.9)		
Health literacy		0–12	7.9 ± 3.5
Inadequate (≤6)	78 (28.1)		
Marginal (7–10)	126 (45.5)		
Adequate (11–12)	73 (26.4)		
Antithrombotic therapy		3–6	5.3 ± 0.8
Non-adherence (1–5)	139 (50.2)		
Adherence (6)	138 (49.8)		

SD, standard deviation.

**Table 3:** Differences in adherence to antithrombotic therapy according to patients’ characteristics, cognitive function, and health literacy (*n* = 277).

Characteristics	Non-Adherence (*n* = 139)	Adherence (*n* = 138)	χ^2^ (*p*)
	Frequency (%)	Frequency (%)	
Age (years)			
65–70	31 (34.8)	58 (65.2)	15.15 (0.001)
70–79	67 (53.2)	59 (46.8)	
≥80	41 (66.1)	21 (33.9)	
Gender			
Men	76 (46.3)	88 (53.7)	2.37 (0.124)
Women	63 (55.8)	50 (44.2)	
Education			
Below elementary school	63 (66.3)	32 (33.7)	16.18 (0.001)
Middle school	32 (46.4)	37 (53.6)	
High school	31 (40.3)	46 (59.7)	
College	13 (36.1)	23 (63.9)	
Job, yes	21 (36.2)	37 (63.8)	5.73 (0.017)
Spouse, yes	83 (43.7)	107 (56.3)	10.21 (0.001)
Years after diagnosis of AF			
1–2	20 (50.0)	20 (50.0)	0.01 (0.953)
3–4	22 (52.4)	20 (47.6)	
≥5	97(69.8)	98 (50.3)	
Type of AF			
Paroxysmal	79 (46.5)	91 (53.5)	3.70 (0.296)
Persistent and permanent	60 (56.7)	47 (43.3)	
CHA_2_DS_2_-VASc score			
1	5 (23.8)	16 (76.2)	6.32 (0.012)
≥2	134 (52.3)	122 (47.7)	
HAS-BLED score			
1–2	24 (45.3)	29 (54.7)	0.63 (0.428)
≥3	115 (51.3)	109 (48.7)	
Hypertension, yes	111 (50.2)	110 (49.8)	0.01 (0.976)
Diabetes, yes	43 (54.4)	36 (45.6)	0.79 (0.372)
Coronary artery disease, yes	46 (51.1)	44 (48.9)	0.05 (0.830)
Heart failure, yes	60 (54.5)	50 (45.5)	1.39 (0.238)
Stroke, yes	31 (54.4)	26 (45.6)	0.51 (0.476)
Aspirin, yes	55 (45.1)	67 (54.9)	2.27 (0.132)
Warfarin, yes	46 (46.5)	53 (53.5)	0.95 (0.356)
NOACs, yes	47 (59.5)	32 (40.5)	3.83 (0.051)
Cognitive function			
Impaired	58 (72.5)	22 (27.5)	22.42 (<0.001)
Normal	81 (41.1)	116 (58.9)	
Health literacy			
Inadequate	56 (71.9)	22 (28.2)	22.00 (<0.001)
Marginal	57 (45.2)	69 (54.8)	
Adequate	26 (35.6)	47 (64.4)	

AF, atrial fibrillation; NOAC, non-vitamin K antagonist oral anticoagulants.

**Table 4:** Factors influencing non-adherence to antithrombotic therapy in patients with atrial fibrillation (*n* = 277).

Variables	Categories	Univariate		Multivariate	
		OR (95% CI)	*p*	OR (95% CI)	*p*
Age (year)	<80	1		1	
	≤79	2.33 (1.291–4.207)	0.005	1.24 (0.621–2.459)	0.546
Education	Above high school	1		1	
	Below middle school	2.17 (1.332–3.549)	0.002	1.45 (0.843–2.493)	0.179
Job	Yes	1		1	
	No	2.06 (1.132–3.742)	0.018	1.29 (0.660–2.522)	0.457
Spouse	Yes	1		1	
	No	2.33 (1.379–3.933)	0.002	1.51 (0.830–2.737)	0.178
CHA_2_DS_2_-VASc score	1	1		1	
	≥2	3.52 (1.250–9.882)	0.017	1.75 (0.573–5.356)	0.326
Cognitive function	Normal	1		1	
	Impaired	3.78 (2.142–6.656)	<0.001	2.63 (1.424–4.848)	0.002
Health literacy	Adequate	1		1	
	Marginal and inadequate	2.25 (1.291–3.902)	0.004	1.45 (0.790–2.644)	0.232

OR, odds ratio; CI, confidence interval.
